# Previous incarceration impacts access to hepatitis C virus (HCV) treatment among HIV‐HCV co‐infected patients in Canada

**DOI:** 10.1002/jia2.25197

**Published:** 2018-11-21

**Authors:** Nadine Kronfli, Roy Nitulescu, Joseph Cox, Erica EM Moodie, Alexander Wong, Curtis Cooper, John Gill, Sharon Walmsley, Valérie Martel‐Laferrière, Mark W Hull, Marina B Klein, the Canadian Co‐Infection Cohort Study

**Affiliations:** ^1^ Division of Infectious Diseases/Chronic Viral Illness Service Department of Medicine Glen site McGill University Health Centre Montreal QC Canada; ^2^ Research Institute of the McGill University Health Centre Montreal QC Canada; ^3^ Department of Epidemiology, Biostatistics, and Occupational Health McGill University Montreal QC Canada; ^4^ Department of Medicine University of Saskatchewan Regina SK Canada; ^5^ Department of Medicine University of Ottawa Ottawa ON Canada; ^6^ Department of Medicine University of Calgary Calgary AB Canada; ^7^ University Health Network Toronto ON Canada; ^8^ CIHR Canadian HIV Trials Network Vancouver BC Canada; ^9^ Centre de Recherche du Centre Hospitalier de l’ Université de Montréal Montreal QC Canada; ^10^ Department of Medicine University of British Columbia Vancouver BC Canada; ^11^ British Columbia Centre for Excellence in HIV/AIDS Vancouver BC Canada

**Keywords:** HIV‐hepatitis C co‐infection, direct‐acting antivirals, people in prison, micro‐elimination, HCV elimination, disparities

## Abstract

**Introduction:**

The prevalence of hepatitis C virus (HCV) is far higher in prison settings than in the general population; thus, micro‐elimination strategies must target people in prison to eliminate HCV. We aimed to examine incarceration patterns and determine whether incarceration impacts HCV treatment uptake among Canadian HIV‐HCV co‐infected individuals in the direct‐acting antiviral (DAA) era.

**Methods:**

The Canadian Co‐Infection Cohort prospectively follows HIV‐HCV co‐infected people from 18 centres. HCV RNA‐positive participants with available baseline information on incarceration history were included and followed from 21 November 2013 (when second‐generation DAAs were approved by Health Canada) until 30 June 2017. A Cox proportional hazards model was used to assess the effect of time‐updated incarceration status on time to treatment uptake, adjusting for patient‐level characteristics known to be associated with treatment uptake in the DAA era.

**Results:**

Overall, 1433 participants (1032/72% men) were included; 67% had a history of incarceration and 39% were re‐incarcerated at least once. Compared to those never incarcerated, previously incarcerated participants were more likely to be Indigenous, earn <$1500 CAD/month, report current or past injection drug use and have poorly controlled HIV. There were 339 second‐generation DAA treatment initiations during follow‐up (18/100 person‐years). Overall, 48% of participants never incarcerated were treated (27/100 person‐years) compared to only 31% of previously incarcerated participants (15/100 person‐years). Sustained virologic response (SVR) rates at 12 weeks were 95% and 92% respectively. After adjusting for other factors, participants with a history of incarceration (adjusted hazard ratio (aHR): 0.7, 95% CI: 0.5 to 0.9) were less likely to initiate treatment, as were those with a monthly income <$1500 (aHR: 0.7, 95% CI: 0.5 to 0.9) or who reported current injection drug use (aHR: 0.7, 95% CI: 0.4 to 1.0). Participants with undetectable HIV RNA (aHR: 2.1, 95% CI: 1.6 to 2.9) or significant fibrosis (aHR: 1.5, 95% CI: 1.2 to 1.9) were more likely to initiate treatment.

**Conclusions:**

The majority of HIV‐HCV co‐infected persons had a history of incarceration. Those previously incarcerated were 30% less likely to access treatment in the DAA era even after accounting for several patient‐level characteristics. With SVR rates above 90%, HCV elimination may be possible if treatment is expanded for this vulnerable and neglected group.

## 1 Introduction

In light of significant advances in combination antiretroviral therapy (cART) resulting in dramatic reductions in AIDS‐related morbidity and mortality, liver disease has emerged as the leading cause of death among people living with HIV primarily due to hepatitis C virus (HCV) co‐infection 1, 2, 3. Due to shared routes of transmission, global estimates indicate that 2.3 million people are co‐infected with HIV and HCV, with the greatest burden in eastern Europe and central Asia followed by sub‐Saharan Africa 4. Worldwide, approximately 60% of co‐infected people have injected drugs, many of whom have spent time in some form of correctional facility during their lifetimes 4. Several HCV‐mono and HIV‐HCV co‐infected sub‐populations, including people who inject drugs (PWID), have failed to benefit from treatment expansion efforts despite being disproportionately affected 5, 6. Given the heterogeneity of those infected by HCV, experts are encouraging the “micro‐elimination” of HCV, whereby specific and effective treatment interventions are directed towards individual sub‐populations such as PWID or people in prison 7.

Due to a high lifetime prevalence of injection drug use (IDU), incarcerated populations are disproportionately burdened by chronic HCV 8. Approximately one‐third of the 11 million people imprisoned worldwide at any given time have been previously exposed to HCV, with differences in country‐level estimates related primarily to geography and prevalence of IDU 9, 10. In the United States, the correctional population represents one‐third of all national HCV cases 11, underscoring the importance of systematic HCV screening, improved linkage to HCV care following release and expanded treatment efforts within and outside prison settings.

Currently, limited data exist on linkage to care and treatment initiation in the direct‐acting antiviral (DAA) era for HCV‐mono‐ or HIV‐HCV co‐infected individuals from correctional facilities outside the United States 12, 13. Given the important contribution of incarceration on perpetuating the HCV epidemic 14 and the availability of curative DAA therapy, prioritizing the treatment of people in and recently released from prison with chronic HCV will be essential to achieve the 2030 HCV elimination goals set by the World Health Organization 15. The aim of this study was to examine incarceration patterns among HIV‐HCV co‐infected persons in Canada and to determine whether a history of incarceration impacts HCV treatment uptake in the DAA era.

## 2 Methods

### 2.1 Study population

We used data from the Canadian Co‐infection Cohort Study (CCC; CTN222), a prospective multicentre study recruiting patients 16 years of age and older with documented HIV infection (HIV seropositive by enzyme‐linked immunosorbent assay (ELISA) with western blot confirmation) and with chronic HCV infection or evidence of HCV exposure (e.g. HCV seropositive by ELISA with recombinant immunoblot assay II or enzyme immunoassay confirmation, or if serologically false‐negative, HCV RNA positive). From April 2003 to 30 June 2017, 1788 patients were enrolled from 18 sites across six Canadian provinces. Participating centres included large urban tertiary care hospitals, community‐based HIV clinics and street outreach programmes in urban and semi‐urban settings in an attempt to capture a representative population of co‐infected patients in care. All eligible patients were approached to participate to avoid selection bias. Cohort design and protocol have been reported in detail elsewhere 16.

### 2.2 Data collection

After written informed consent was obtained, patients underwent an initial evaluation followed by study visits approximately every six months. At each visit, sociodemographic and behavioural information (including substance use, health services utilization and incarceration) were self‐reported in questionnaires, medical treatments and diagnoses were collected by research personnel, and laboratory analyses were performed. The study was approved by the community advisory committee of the Canadian Institutes of Health Research (CIHR)‐Canadian HIV Trials Network and by all institutional ethics boards of participating centres.

### 2.3 Statistical analyses

#### 2.3.1 Incarceration patterns

In order to assess incarceration patterns, we selected participants who had available information on history of incarceration at enrolment and at least two cohort visits. We compared baseline sociodemographic, behavioural and clinical characteristics between patients with and without a history of incarceration at enrolment. Comparisons were made using a Fisher's exact test for binary variables, a chi‐squared test for categorical variables and a Wilcoxon rank‐sum test for continuous variables.

Time to incarceration during study follow‐up was assessed separately among patients with and without a history of incarceration at enrolment using the Kaplan–Meier method and a comparison was made using a log‐rank test. Eligible patients were followed from enrolment until they first became incarcerated during follow‐up. Patients who were never incarcerated during the study period were censored at death, loss to follow‐up (no visits for more than 1.5 years), withdrawal of consent or at administrative censoring on 30 June 2017, whichever occurred first. Rates of incarceration and median time to incarceration were reported with their 95% confidence intervals (CI).

#### 2.3.2 Treatment uptake

Second‐generation DAAs (starting with simeprevir) were first approved by Health Canada on 21 November 2013. Access to second‐generation DAAs according to incarceration history was assessed among a subgroup of patients who: (1) had available information on history of incarceration at enrolment, (2) were HCV RNA positive on or after 21 November 2013 and (3) did not die, withdraw consent, become lost to follow‐up, successfully cure their HCV infection through treatment or initiate a second‐generation DAA prior to 21 November 2013.

Treatment initiations were considered eligible outcomes if they contained any of the following: simeprevir, sofosbuvir, ledipasvir, velpatasvir, ombitasvir/paritaprevir/ritonavir, daclatasvir, grazoprevir or elbasvir.

Patients were followed‐up from 21 November 2013 or upon enrolment into the cohort, whichever occurred later. Follow‐up ended if an eligible treatment was initiated or if patients were censored. Censoring was applied at the earliest date that any of the following occurred: (1) spontaneous clearance of HCV, (2) death, (3) withdrawal of consent, (4) loss to follow‐up, (5) initiation of a treatment that did not contain a second‐generation DAA or (6) administrative censoring on 30 June 2017.

Time to DAA treatment uptake was modelled with a multivariate Cox proportional hazards model using robust standard errors. The exposure of interest was time‐updated incarceration history. The following adjustment covariates, known to be associated with treatment uptake in the DAA era, were chosen a priori and measured at the cohort visit closest to the start of study follow‐up: age, sex, Indigenous ethnicity, monthly income (≤1500 Canadian dollars (CAD)), a history of IDU, current IDU (within the past six months), hazardous drinking in the past six months (as defined by the AUDIT‐C 17), history of psychiatric diagnosis (depression, bipolar disorder, schizophrenia, personality disorder) or hospitalization, HCV genotype 3, advanced liver fibrosis (based on an aspartate‐to‐platelet ratio index (APRI) greater than 1.5 at any time prior to the start of study follow‐up), undetectable HIV viral load (≤50 copies/mL) and Canadian province. When adjusting for province, British Columbia was used as the reference with individual indicators for Saskatchewan and Quebec, and a combined indicator for Ontario, Alberta and Nova Scotia. This reflects the regional differences in criteria for access to, and reimbursement of, DAA therapies for co‐infected patients during the study period. Specifically, reimbursement criteria based on the level of liver fibrosis varied across provinces with Quebec having the most liberal policies 18. All analyses were conducted using R statistical software 19.

## 3 Results

### 3.1 Participant sociodemographic and clinical characteristics

A total of 1433 HIV‐HCV co‐infected patients were included following the exclusion of those with missing baseline incarceration information (n = 107) and those with fewer than two cohort visits (e.g. recently enrolled; n = 248). Of those remaining, 67% (955/1433) had a history of previous incarceration. Patient sociodemographic, behavioural and clinical characteristics stratified by incarceration history are presented in Table 1. Compared to those who were never incarcerated, previously incarcerated patients were younger, more likely to report Indigenous ethnicity, earn less than $1500 CAD per month, be homeless or live in a shelter, and report current or a history of IDU and current use of other drugs. With respect to HIV infection, those with a history of incarceration were less likely to be on cART and be virally suppressed, and were more likely to have a lower median CD4+ T‐cell count. Regarding HCV infection, patients with a history of incarceration were more likely to have genotype 3 infection and have longer durations of infection despite similar proportions of advanced liver fibrosis. Those previously incarcerated also reported more frequent use of healthcare services. Furthermore, 23% reported IDU and 23% having had a tattoo done while in prison.

**Table 1:** Comparison of baseline characteristics between patients with and without a history of incarceration at enrolment

Characteristic at cohort enrolment	History of incarceration n = 955	No history of incarceration n = 478	*p*‐value
Age (years), median (Q1; Q3)	44 (38; 50)	47 (41; 52)	<0.001
Female	29%	26%	0.318
Indigenous	26%	13%	<0.001
Province	–	–	<0.001
Alberta	3%	3%	
British Columbia	30%	25%	
Nova Scotia	1%	1%	
Ontario	20%	31%	
Quebec	35%	35%	
Saskatchewan	11%	5%	
Monthly income ≤ $1500 CAD	85%	60%	<0.001
Homeless or living in a shelter or residence	15%	5%	<0.001
History of drug addiction therapy	76%	35%	<0.001
History of injection drug use	94%	57%	<0.001
Current injection drug use^a^	46%	20%	<0.001
Current non‐injection drug use^a^	50%	35%	<0.001
Current marijuana use^a^	56%	46%	<0.001
HCV duration (years), median (Q1; Q3)	11 (6; 16)	9 (2; 16)	<0.001
HCV treatment naïve	84%	73%	<0.001
HCV genotype	–	–	<0.001
Genotype 1	59%	67%	
Genotype 2	3%	5%	
Genotype 3	19%	13%	
Genotype 4	1%	6%	
Genotype unknown	17%	9%	
APRI > 1.5	18%	21%	0.114
Prior diagnosis of end‐stage liver disease^b^	8%	11%	0.041
HIV viral load ≤50 copies/mL	61%	70%	<0.001
On antiretroviral therapy	84%	89%	0.009
CD4 cell count, median (Q1; Q3)	390 (240; 570)	430 (260; 600)	0.014
Health service utilization in past 6 months, mean (standard deviation)	–	–	–
Number of emergency room visits	1.2 (4.2)	0.7 (1.9)	0.005
Number of overnight hospitalizations	1.5 (8.1)	0.6 (2.6)	0.033
History of injection drug use in prison	23%		
History of tattoo in prison	23%		
History of body piercing in prison	3%		

APRI, aspartate‐to‐platelet ratio index; CAD, Canadian dollars; HCV, hepatitis C virus; Q1, first quartile of distribution; Q3, third quartile of distribution.

^a^ Defined as within the last six months; ^b^diagnosis of ascites, cirrhosis, portal hypertension, spontaneous bacterial peritonitis, encephalopathy, oesophageal varices, hepatocellular carcinoma or hepatorenal syndrome.

### 3.2 Incarceration patterns

Among the 955 patients with a history of incarceration, 368 (39%) were re‐incarcerated at least once during follow‐up, with an incidence rate for first re‐incarceration of 11.3 per 100 person‐years (95% CI: 10.2 to 12.5). In contrast, among the 478 patients with no history of incarceration, 35 (7%) were incarcerated during follow‐up, with an incidence rate for first incarceration of 1.6 per 100 person‐years (95% CI: 1.1 to 2.2).

**Figure 1:**
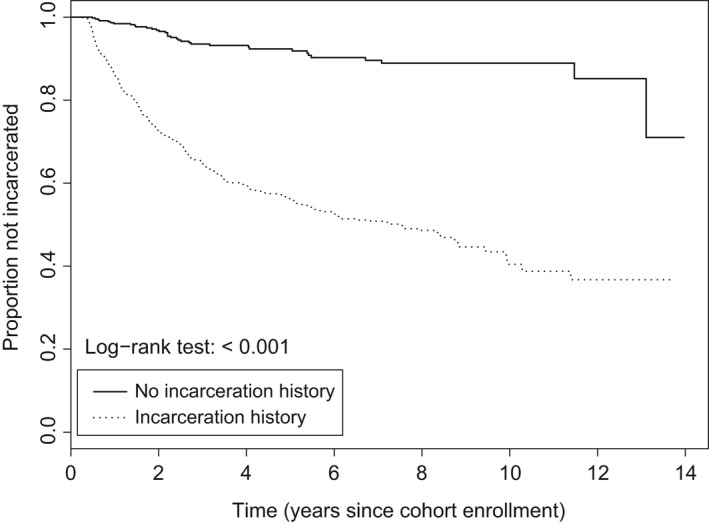
Kaplan–Meier plot of time to incarceration, stratified by incarceration history. Probability of not being incarcerated at any time since entry into the Canadian Co‐infection Cohort.

Figure**** 1 shows the Kaplan–Meier survival curves for time to incarceration stratified by incarceration history. Patients with a history of incarceration were significantly more likely to be incarcerated during follow‐up than those without a history of incarceration. The median time to re‐incarceration among those previously incarcerated was 7.5 years (95% CI: 5.5 to 8.9).

### 3.3 Treatment uptake

**Figure 2:**
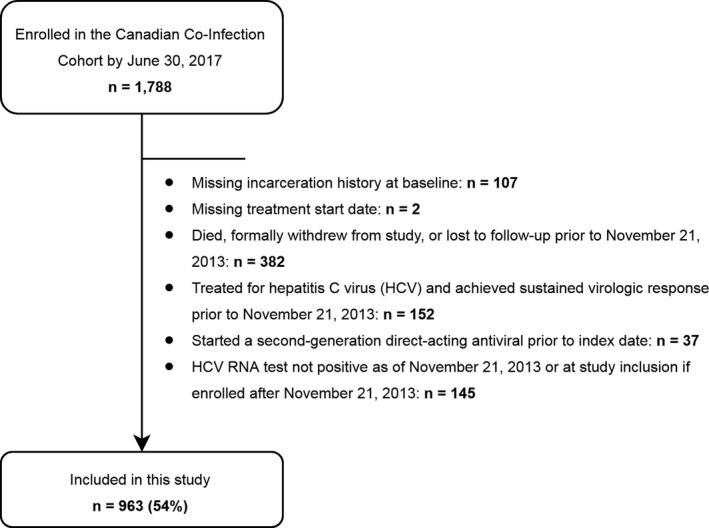
Sample selection flow chart. Number of patients selected for the analysis of treatment uptake during the DAA era and number excluded by criteria.

A total of 963 (54%) cohort participants met all eligibility criteria for the analysis of time to DAA uptake (Figure 2). During follow‐up, 339 patients started an eligible second‐generation DAA treatment course (18 treatments per 100 person‐years), of which 96% were interferon‐free. The remaining patients were censored due to loss to follow‐up (n = 175), death (n = 50), the initiation of a treatment that did not contain a second‐generation DAA (n = 17), spontaneous clearance of HCV (n = 16), withdrawal of consent (n = 11) or administrative censoring (n = 355).

Overall, 48% (125/263) of participants with no history of incarceration were treated (27 treatments per 100 person‐years) compared to 31% (214/700) of previously incarcerated participants (15 treatments per 100 person‐years). Sustained virologic response (SVR) rates at 12 weeks were 95% and 92% respectively.

Table 2 presents the adjusted hazard ratio (aHR) estimates from the analysis of time to treatment uptake. Independent of other factors included in the multivariable model, time‐updated incarceration was associated with a lower risk of treatment initiation (aHR: 0.7, 95% CI: 0.5 to 0.9). Other factors associated with lower risk of treatment uptake included IDU in the last six months, a monthly income of less than $1500 CAD and residency in Saskatchewan (compared to residency in the province of British Columbia). Patients with advanced fibrosis (APRI > 1.5), undetectable HIV viral loads and residency in Quebec were more likely to be treated for HCV. There was no evidence of effect modification by sex (interaction term between sex and incarceration status (aHR: 1.1, 95% CI: 0.6 to 2.0)).

**Table 2:** Multivariate Cox proportional hazards time‐to‐event model for direct‐acting antiviral treatment uptake (n = 964)

Covariate at beginning of study follow‐up^b^	Adjusted hazard ratio (95% confidence interval)
Time‐updated incarceration history	0.7 (0.5; 0.9)
Age (per 10 years)	1.1 (0.9; 1.2)
Female	0.9 (0.7; 1.1)
Indigenous	0.8 (0.6; 1.2)
History of injection drug use	0.9 (0.7; 1.3)
Current injection drug use^a^	0.7 (0.4; 1.0)
Current hazardous drinking^a^^**,**^ c	0.9 (0.7; 1.2)
HCV genotype 3	0.8 (0.6; 1.1)
APRI > 1.5^d^	1.5 (1.2; 1.9)
HIV viral load ≤ 50 copies/mL	2.1 (1.6; 2.9)
History of psychiatric diagnosis or hospitalizations^e^	0.9 (0.7; 1.1)
Monthly income ≤ $1500 CAD	0.7 (0.5; 0.9)
British Columbia (reference)	1.0
Quebec	1.5 (1.1; 2.0)
Saskatchewan	0.2 (0.1; 0.5)
Ontario, Alberta and Nova Scotia	0.9 (0.7; 1.3)

APRI, aspartate‐to‐platelet ratio index; CAD, Canadian dollars; HCV, hepatitis C virus

^a^Defined as within the last six months; ^b^measured at the cohort visit closest to the beginning of study follow‐up; ^c^hazardous drinking was defined as an AUDIT‐C score of at least 4 for males and at least 3 for females; ^d^as measured at any time prior to the beginning of study follow‐up; ^e^psychiatric diagnoses comprised depression, bipolar disorder, schizophrenia and personality disorder.

## 4 Discussion

Our study offers the first description of incarceration patterns and the effects of incarceration on HCV treatment uptake in a large HIV‐HCV co‐infected cohort. Not only was the majority (67%) of our cohort previously incarcerated, but we observed a high re‐incarceration incidence rate. Results from our study also provide evidence that previous incarceration is an important patient‐level barrier to HCV treatment initiation in the DAA era among HIV‐HCV co‐infected persons in Canada even after accounting for several patient‐level characteristics. While it is probable that the high re‐incarceration rates may have impacted treatment initiation, it is possible that other unmeasured social determinants or behavioural attributes among those with a history of incarceration (e.g. mistrust in the health system, psychological distress or food and housing insecurities) may have contributed to the observed lower rates of treatment. This is despite engagement of HIV‐HCV co‐infected populations in HIV care, facilitating their identification for HCV treatment, and the absence of restrictions for DAA uptake for co‐infected persons based on sociodemographic or behavioural risk factors in Canada 18. In addition to increased interactions with the correctional system, we found that previously incarcerated HIV‐HCV persons have increased urgent medical care visits. These frequent interactions with both correctional services and healthcare systems represent missed opportunities for linkage to HCV care.

While strategies aimed at increasing access to HCV treatment should be explored for people in prison with chronic HCV 20, simultaneously, several factors including high turnover rates owing to short incarcerations, frequent prison transfers and the high cost of DAAs require consideration before treatment is initiated 21. We found equivalently high SVR rates among those with or without an incarceration history, suggesting that the decision to initiate treatment for individuals with a history of incarceration should not be based on a provider's perceived risk of treatment failure. Several countries including Canada, the United States and Australia have recently begun to prioritize treatment of inmates with sentences that allow for the completion of DAA therapy during incarceration 22. This is a reasonable approach owing to lower SVR rates among inmates who are initiated on treatment but who are subsequently transferred or released 23. Given the recent prioritization of HCV treatment for people in federal prisons in Canada 22, where sentences are greater than two years, the results of our study – that HIV‐HCV co‐infected persons with a history of incarceration experience decreased HCV treatment uptake – likely reflect both deficiencies in HCV treatment programmes in Canadian correctional facilities and a lack of linkage to HCV care at the time of release.

Strengthening linkage to HCV care at the time of release is of paramount importance if micro‐elimination of HCV is to occur among people in prison. Recent HCV cascade analyses demonstrate that linkage to care rates following release vary between 9 and 33% in the United States 12, 13, 24, implying that linkage is the rate‐limiting step for treatment uptake for many people in prison with chronic HCV. Interestingly, Hochstatter *et al*. found that released inmates were more likely to link to care if they received *any* HCV care while incarcerated 12. This echoes findings that prison‐based multidisciplinary care is associated with improved engagement along the HCV care cascade and patient‐reported outcomes 25, 26. These results have important implications on prison care strategies and suggest that if such strategies are provided by one or more members of an on‐site multidisciplinary care team, linkage can be improved with minimal costs to the system 21. Furthermore, a recent systematic review evaluating interventions to increase HCV engagement for people in prison found only one study that aimed to improve linkage following the release of inmates 27, highlighting the need for rigorous controlled trials with novel strategies for linkage to care in the DAA era. While linkage to care at the time of release may be particularly challenging for released inmates due to multiple competing priorities 28, 29, several recent studies have demonstrated the feasibility of HCV linkage to care post‐release programmes 13, 24. Although standard procedures to facilitate linkage with care post‐release do not exist in the majority of correctional facilities worldwide, emerging studies suggest that post‐release linkage programmes may succeed if integrated into prison care strategies.

Our results highlight other missed opportunities for linkage to HCV care for persons with a history of incarceration. While we have already emphasized that any interaction with the correctional system should serve as an opportunity for linkage at the time of discharge, interactions with the overall healthcare system should serve a similar purpose. Our study found that HIV‐HCV co‐infected persons with a history of incarceration had twice the number of emergency department (ED) visits and hospitalizations compared to those never incarcerated. Linkage to HCV care for ED patients with known chronic HCV can be challenging for various reasons including that EDs are rarely well integrated into the greater healthcare system. That said, ensuring linkage to care for this population has the potential to decrease incident and prevalent HCV infections 30. A recent study evaluating the HCV cascade of care among those screened for HCV in two EDs in the United States found that 61% of those who had a follow‐up appointment scheduled for HCV care in the ED were subsequently linked to care 31. While these patients were not restricted to those with an incarceration history, this study suggests that linkage to HCV care is feasible following a brief interaction with the healthcare system. A similar screening and linkage programme implemented with baby boomers in a safety net hospital in the United States found that more than 80% of patients were linked to follow‐up HCV care 32, reinforcing that brief or extended interactions with the healthcare system can serve as important opportunities for linkage to HCV care.

While we have specifically emphasized linkage to HCV care at the time of release, in the context of a population with multimorbidity and significant social vulnerabilities, strengthening linkages with primary care rather than disease‐specific specialty care may be the ideal long‐term solution 21. In order for those previously incarcerated to benefit from any healthcare, addressing the social determinants of health becomes particularly valuable; alleviating food and housing insecurities and facilitating employment and other income opportunities, while simultaneously ensuring access to harm reduction services at the time of release undoubtedly takes precedence for many. While primary care may be suited to address some of these challenges, post‐incarceration transitions clinics have also emerged as models of care to address these specific barriers in a culturally appropriate manner 33, 34. By addressing these basic human needs together with HCV care, overall health and quality of life outcomes may improve.

Although a small proportion of correctional facilities have begun to expand prison‐based linkage and treatment programmes, the majority have not yet succeeded in instituting systematic screening programmes despite long‐standing WHO recommendations 35. While our results are likely generalizable to many resource‐constrained settings, HCV linkage and treatment programmes for inmates at the time of release should unlikely be prioritized if systematic screening of high‐risk groups is not yet in place. In order to first expand screening, resource‐limited countries should evaluate the opportunities and challenges of integrated versus vertical care models for HCV diagnosis in services such as HIV clinics, prison health services, and needle syringe and opioid substitution therapy programmes in order to prioritize those incarcerated or who may eventually become incarcerated 36. Scaling up community‐level HCV treatment and care, as prison‐based HCV treatment may unlikely be provided for some time, will then require many intersecting initiatives such as negotiating price reductions, simplifying care, decentralizing care to non‐specialists to overcome human resource constraints, encouraging patient and community engagement, and increasing financial and political commitment 36. As commitments to eliminate HCV begin to rollout in many developed and developing countries, prioritizing an HCV care package for vulnerable groups such as people in prison will be an essential part of the response.

The Canadian Co‐infection Cohort comprises a diverse patient population followed at various primary and tertiary care clinics in urban and semi‐urban areas in Canada and is thus representative of the co‐infected Canadian population 16. However, our study has limitations. We were unable to stratify our data based on the type of Canadian correctional facility, federal or provincial/territorial prisons. However, the Correctional Service of Canada announced that all federal inmates with chronic HCV would be eligible for HCV treatment in July 2017 22, after our study period closed. To account for this policy change, information on type of correctional facility began to be collected in the cohort in April 2018. Another limitation is that the exact dates of incarceration and release were not known. Consequently, when measuring the time to incarceration, we could only use a proxy date for the incarceration event; namely, the date of the cohort visit at which the patient reported being incarcerated in the previous six months. Furthermore, for the same reason, it was not possible to assess the rate of DAA treatment while patients were incarcerated. Finally, our results are not generalizable to HIV‐HCV co‐infected individuals who do not access HIV care; namely, those who are not diagnosed or linked to care, representing approximately 15% and 10% respectively of the HIV‐HCV Canadian co‐infected population 37.

## 5 Conclusions

In order to eliminate HCV by 2030, people in and recently released from prison must be part of the global elimination agenda. Our study identified previous incarceration as an important patient‐level barrier to HCV treatment initiation in the DAA era among HIV‐HCV co‐infected persons in Canada. Until HCV care and treatment programmes become fully integrated in correctional facilities, an emphasis should be made on strengthening linkage to HCV care from incarceration or within the healthcare system itself.

## Competing interests

The authors have no conflict of interest with regard to this study and there was no pharmaceutical industry support to conduct this study. Roy Nitulescu and Erica EM Moodie have no conflicts of interest to declare. Nadine Kronfli has received consulting fees from ViiV Healthcare, Merck and Gilead; research funding from ViiV Healthcare and Gilead; and payment for lectures from Gilead. Joseph Cox has received consulting fees from ViiV Healthcare and Gilead; research funding from ViiV Healthcare, Merck and Gilead; and payment for lectures from Gilead. Alexander Wong has received consulting and honoraria from Merck, Gilead Sciences, Bristol‐Myers Squibb, Pfizer, Janssen, Boehringer‐Ingelheim and AbbVie, and funding for regional and provincial programming from Merck, Gilead Sciences, Bristol‐Myers Squibb, ViiV, Janssen and AbbVie. Curtis Cooper reports consulting fees from AbbVie, Gilead and Merck; and grants from AbbVie and Gilead. John Gill is an ad hoc member of the National HIV advisory Boards for Merck, ViiV Healthcare and Gilead. Sharon Walmsley has received grants, consulting fees, lecture fees, nonfinancial support and fees for the development of educational presentations from Merck, ViiV Healthcare, GlaxoSmithKline, Pfizer, Gilead, Abbvie, Bristol‐Myers Squibb and Janssen. Valérie Martel‐Laferrière has received consulting fees from Merck and Gilead; grants from Gilead; and lecture fees from AbbVie, Merck and Gilead. Mark W. Hull has received honoraria for speaking engagements and/or consulting fees from AbbVie, Bristol‐Myers Squibb, Gilead, Merck, Ortho‐Janssen and ViiV Healthcare. Marina B. Klein has received consulting fees from ViiV Healthcare, BMS, Merck, Gilead and AbbVie; and research funding from Merck, Gilead and ViiV Healthcare.

## Authors’ contributions

As the corresponding author, NK has had full access to all the data in the study and takes responsibility for the integrity of the data and the accuracy of the data analysis. NK and MK conceptualized and designed the study; NK, MK, RN, JC, EM, AW, CC, JG, SW, VLM and MH acquired, analysed or interpreted the data and critically reviewed the manuscript for important intellectual content; NK drafted the manuscript; RN statistically analysed the data.
